# *trans*-Di­fluorido­tetra­kis­(pyridine-κ*N*)chromium(III) perchlorate from synchrotron radiation

**DOI:** 10.1107/S1600536813023052

**Published:** 2013-08-23

**Authors:** Dohyun Moon, Jong-Ha Choi

**Affiliations:** a Pohang Accelerator Laboratory, POSTECH, Pohang 790-784, Republic of Korea; b Department of Chemistry, Andong National University, Andong 760-749, Republic of Korea

## Abstract

The are two independent complex cations in the title salt, [CrF_2_(C_5_H_5_N)_4_]ClO_4_, each located on a centre of inversion, as well as an independent perchlorate counter-ion. The complex cations adopt slightly distorted octa­hedral coordination environments around the Cr^III^ ion, defined by four pyridine (py) N atoms in the equatorial plane and two F^−^ ligands in the axial positions; intra­molecular C—H⋯F contacts are noted. The mean Cr—N(py) and Cr—F bond lengths are 2.088 (6) and 1.8559 (10) Å, respectively. The three-dimensional architecture is sustained by hydrogen bonds involving the pyridine C—H groups as donors, and F and O atoms as acceptors.

## Related literature

For background to geometric isomerism in transition metal comlexes, see: Knight & Scott (2003 ▶); Ronconi & Sadler (2007 ▶). For the synthesis, see: Glerup *et al.* (1970 ▶). For the structure of *trans*-[Cr(py)_4_F_2_]PF_6_, see: Fochi *et al.* (1991 ▶).
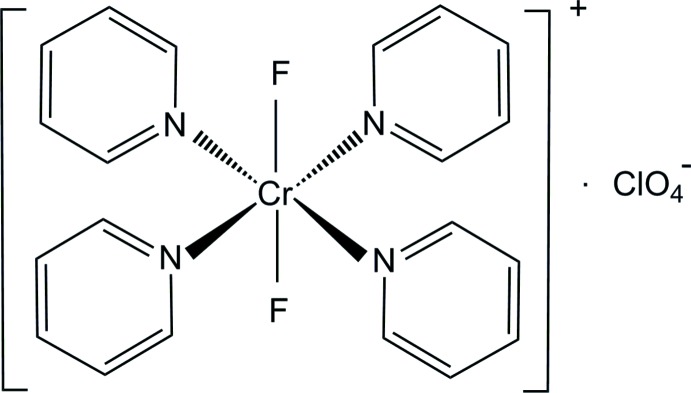


## Experimental

### 

#### Crystal data

[CrF_2_(C_5_H_5_N)_4_]ClO_4_
*M* *_r_* = 505.85
Triclinic, 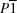

*a* = 9.5690 (19) Å
*b* = 10.615 (2) Å
*c* = 12.663 (3) Å
α = 68.46 (3)°
β = 68.31 (3)°
γ = 79.38 (3)°
*V* = 1109.9 (5) Å^3^
*Z* = 2
Synchrotron radiation
λ = 0.63001 Å
μ = 0.49 mm^−1^
*T* = 99 K
0.03 × 0.03 × 0.02 mm

#### Data collection

ADSC Q210 CCD area-detector
Absorption correction: empirical (*HKL3000sm*; Otwinowski & Minor, 1997 ▶) *T*_min_ = 0.985, *T*_max_ = 0.993
11523 measured reflections
5842 independent reflections
4912 reflections with *I* > 2σ(*I*)
*R*_int_ = 0.020

#### Refinement

*R*[*F* ^2^ > 2σ(*F* ^2^)] = 0.036
*wR*(*F* ^2^) = 0.099
*S* = 1.05
5842 reflections
293 parameters
H-atom parameters constrained
Δρ_max_ = 0.51 e Å^−3^
Δρ_min_ = −0.61 e Å^−3^

### 

Data collection: *PAL ADSC Quantum-210 ADX Software* (Arvai & Nielsen, 1983 ▶); cell refinement: *HKL3000sm* (Otwinowski & Minor, 1997 ▶); data reduction: *HKL3000sm*; program(s) used to solve structure: *SHELX-2013-XS* (Sheldrick, 2008 ▶); program(s) used to refine structure: *SHELX-2013-XL* (Sheldrick, 2008 ▶); molecular graphics: *DIAMOND* (Brandenburg, 2007 ▶); software used to prepare material for publication: *WinGX* (Farrugia, 2012 ▶).

## Supplementary Material

Crystal structure: contains datablock(s) I. DOI: 10.1107/S1600536813023052/tk5248sup1.cif

Structure factors: contains datablock(s) I. DOI: 10.1107/S1600536813023052/tk5248Isup2.hkl

Additional supplementary materials:  crystallographic information; 3D view; checkCIF report

## Figures and Tables

**Table 1:** Hydrogen-bond geometry (Å, °)

*D*—H⋯*A*	*D*—H	H⋯*A*	*D*⋯*A*	*D*—H⋯*A*
C1—H1⋯F1	0.95	2.36	2.892 (2)	115
C6—H6⋯F1	0.95	2.31	2.874 (2)	118
C11—H11⋯F2	0.95	2.32	2.879 (2)	117
C16—H16⋯F2	0.95	2.39	2.915 (2)	115
C14—H14⋯O1*P*	0.95	2.61	3.154 (2)	117
C9—H9⋯O2*P*	0.95	2.63	3.320 (3)	130
C1—H1⋯O3*P* ^i^	0.95	2.41	3.107 (2)	130
C4—H4⋯O1*P* ^ii^	0.95	2.51	3.368 (2)	150
C5—H5⋯F1^iii^	0.95	2.38	2.900 (2)	114
C10—H10⋯F1^iii^	0.95	2.31	2.863 (2)	117
C15—H15⋯F2^iv^	0.95	2.29	2.856 (2)	117
C20—H20⋯F2^iv^	0.95	2.34	2.860 (2)	114
C15—H15⋯O3*P* ^v^	0.95	2.64	3.466 (2)	146
C19—H19⋯O2*P* ^vi^	0.95	2.58	3.227 (3)	125

Symmetry codes: (i) 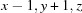; (ii) 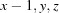; (iii) 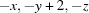; (iv) 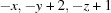; (v) 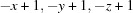; (vi) 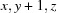.

**Fig. 1.**
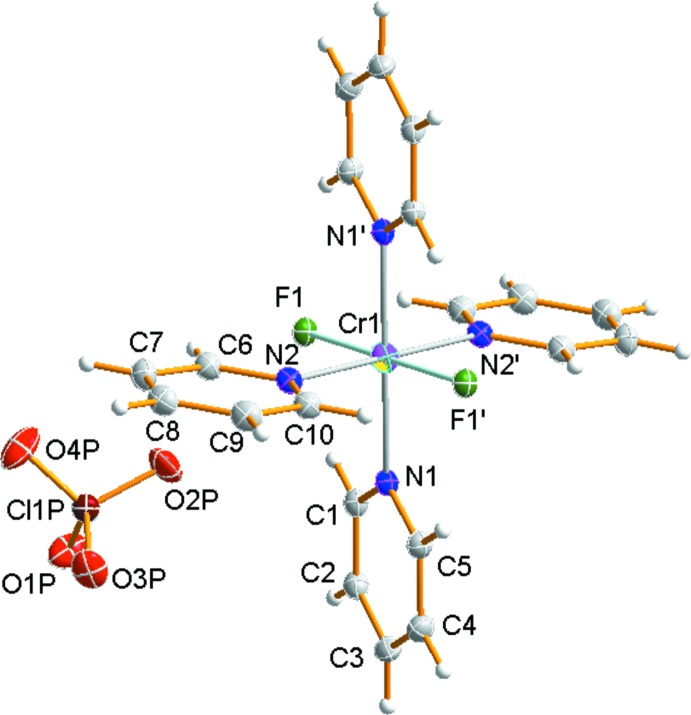
A perspective drawing (50% probability level) of one independent complex cation and the unique perchlorate anion in the structure of trans-[Cr(py)4F2]ClO4

## supplementary crystallographic information

### 1. Comment

The study of geometrical isomerism in octahedral transition metal complexes with mixed ligands has generated considerable interest, and has provided much basic structural information and spectroscopic properties (Knight & Scott, 2003). The geometry of various ligands in the metal complexes are very important in medical applications, and is likely to be a major factor in determining the anti-viral activity and its side-effects (Ronconi & Sadler, 2007). The [Cr(py)_4_X_2_]^n+^ cation (X = monodentate; py = pyridine) can be either *trans* or *cis* geometric isomers. The infrared, electronic absorption and emission spectroscopic properties are useful in distinguishing the geometric isomers of chromium(III) complexes. However, it should be noted that the geometric and conformational assignments based on spectroscopic properties are not always definitive.

In this communication, we describe the structure of *trans*-[Cr(py)_4_F_2_]ClO_4_ in order to confirm the coordination of four pyridine molecules in equatorial plane and two fluoride ligands in axial positions. Counter anionic species play a very important role in coordination chemistry. This is another example of a *trans*-[Cr(py)_4_F_2_]^+^ structure but with a different counter anion.

The structural analysis shows the Cr^III^ complex cation to be coordinated by four nitrogen atoms of four py ligands in the equatorial sites and the two mutually *trans* fluoride atoms. The Cr1 and Cr2 complex cations are in half occupancy in the asymmetric unit. That is, each molecule is contributing a charge of +0.5. Thus, the salt comprises *trans*-[Cr(py)_4_F_2_]^+^ and ClO_4_^-^. An ellipsoid plot of one independent complex cation and the anion is depicted in Fig. 1.

Atoms Cr1 and Cr2 are located at a crystallographic center of symmetry, so these Cr complex cations have molecular *C_i_* symmetry.

The Cr—N(py) distances vary from 2.0799 (17) to 2.0929 (15) Å and the Cr–F distances are in the range of 1.8552 (10) to 1.8566 (10) Å. These bond lengths are in good agreement with those observed in *trans*-[Cr(py)_4_F_2_]PF_6_ (Fochi *et al.*, 1991).

The ClO_4_^-^ anion remains outside the coordination sphere. The crystal packing is stabilized by hydrogen bonding interactions between the C—H groups of the py ligand and the oxygens of the ClO_4_^-^ anion, Table 1. As expected, the ClO_4_^-^ counter ion has slightly distorted tetrahedral geometry due to the influence of hydrogen bonding on the Cl—O bond lengths and the O—Cl–O angles. Consideration of the crystal packing shows that intermolecular C—H···F hydrogen bonds are also present, Table 1.

### 2. Experimental

All chemicals were reagent grade materials and used without further purification. The *trans*-[Cr(py)_4_F_2_]ClO_4_ salt was prepared as described previously (Glerup *et al.*, 1970), and allowed to stand in 0.1 M HClO_4_ solution at room temperature for 1-2 days to give very small crystals suitable for X-ray structural analysis.

### 3. Refinement

C-bound H-atoms were placed in calculated positions (C—H = 0.95) and were included in the refinement in the riding model approximation with *U*_iso_(H) set to 1.2*U*_eq_(C).

### Crystal data

**Table:**

[CrF_2_(C_5_H_5_N)_4_]ClO_4_	*Z* = 2
*M**_r_* = 505.85	*F*(000) = 518
Triclinic, *P*1	*D*_x_ = 1.514 Mg m^−^^3^
Hall symbol: -P 1	Synchrotron radiation, λ = 0.63001 Å
*a* = 9.5690 (19) Å	Cell parameters from 33119 reflections
*b* = 10.615 (2) Å	θ = 0.4–33.6°
*c* = 12.663 (3) Å	µ = 0.49 mm^−^^1^
α = 68.46 (3)°	*T* = 99 K
β = 68.31 (3)°	Pink, plate
γ = 79.38 (3)°	0.03 × 0.03 × 0.02 mm
*V* = 1109.9 (5) Å^3^	

### Data collection

**Table:**

ADSC Q210 CCD area-detector diffractometer	4912 reflections with *I* > 2σ(*I*)
Radiation source: PLSII 2D bending magnet	*R*_int_ = 0.020
ω scan	θ_max_ = 26.0°, θ_min_ = 1.6°
Absorption correction: empirical (using intensity measurements) (*HKL3000sm*; Otwinowski & Minor, 1997)	*h* = −13→13
*T*_min_ = 0.985, *T*_max_ = 0.993	*k* = −14→14
11523 measured reflections	*l* = −17→17
5842 independent reflections	

### Refinement

**Table:**

Refinement on *F*^2^	Hydrogen site location: inferred from neighbouring sites
Least-squares matrix: full	H-atom parameters constrained
*R*[*F*^2^ > 2σ(*F*^2^)] = 0.036	*w* = 1/[σ^2^(*F*_o_^2^) + (0.0543*P*)^2^ + 0.5188*P*] where *P* = (*F*_o_^2^ + 2*F*_c_^2^)/3
*wR*(*F*^2^) = 0.099	(Δ/σ)_max_ = 0.001
*S* = 1.05	Δρ_max_ = 0.51 e Å^−^^3^
5842 reflections	Δρ_min_ = −0.61 e Å^−^^3^
293 parameters	Extinction correction: *SHELXL*, Fc^*^=kFc[1+0.001xFc^2^λ^3^/sin(2θ)]^-1/4^
0 restraints	Extinction coefficient: 0.062 (4)

### Special details

**Table:**

Experimental. Since the Pohang Accelerator Laboratory goniostat has only one omega-axis, diffrn_measured_fraction_theta_full is not fully covered as 0.944, especially for the low symmetry such as a triclinic system. As this is an inherent problem, other command and option (such as *OMIT*) were not helpful to improve the completeness.
Geometry. All e.s.d.'s (except the e.s.d. in the dihedral angle between two l.s. planes) are estimated using the full covariance matrix. The cell e.s.d.'s are taken into account individually in the estimation of e.s.d.'s in distances, angles and torsion angles; correlations between e.s.d.'s in cell parameters are only used when they are defined by crystal symmetry. An approximate (isotropic) treatment of cell e.s.d.'s is used for estimating e.s.d.'s involving l.s. planes.

### Fractional atomic coordinates and isotropic or equivalent isotropic displacement parameters (Å^2^)

**Table:**

	*x*	*y*	*z*	*U*_iso_*/*U*_eq_	
Cr1	0.0000	1.0000	0.0000	0.01263 (9)	
F1	0.01103 (10)	1.14193 (9)	0.04859 (8)	0.01655 (19)	
N1	−0.16449 (15)	0.91455 (13)	0.16450 (12)	0.0144 (2)	
C1	−0.24067 (17)	0.99130 (16)	0.23459 (14)	0.0160 (3)	
H1	−0.2177	1.0836	0.2077	0.019*	
C2	−0.35141 (18)	0.94021 (17)	0.34453 (15)	0.0184 (3)	
H2	−0.4036	0.9969	0.3918	0.022*	
C3	−0.38521 (19)	0.80573 (18)	0.38478 (15)	0.0206 (3)	
H3	−0.4594	0.7682	0.4605	0.025*	
C4	−0.30797 (19)	0.72648 (17)	0.31164 (16)	0.0213 (3)	
H4	−0.3294	0.6341	0.3366	0.026*	
C5	−0.19964 (18)	0.78444 (16)	0.20218 (15)	0.0177 (3)	
H5	−0.1484	0.7307	0.1520	0.021*	
N2	0.17550 (15)	0.89172 (13)	0.06286 (12)	0.0150 (3)	
C6	0.24772 (18)	0.94873 (16)	0.10540 (14)	0.0182 (3)	
H6	0.2131	1.0362	0.1120	0.022*	
C7	0.37130 (19)	0.88404 (18)	0.14009 (16)	0.0212 (3)	
H7	0.4210	0.9271	0.1691	0.025*	
C8	0.42122 (19)	0.75607 (18)	0.13180 (15)	0.0214 (3)	
H8	0.5052	0.7097	0.1556	0.026*	
C9	0.34678 (19)	0.69668 (17)	0.08827 (16)	0.0210 (3)	
H9	0.3789	0.6089	0.0817	0.025*	
C10	0.22513 (18)	0.76705 (16)	0.05457 (15)	0.0181 (3)	
H10	0.1746	0.7262	0.0245	0.022*	
Cr2	0.0000	1.0000	0.5000	0.01280 (9)	
F2	−0.15818 (10)	1.01373 (10)	0.44431 (8)	0.01706 (19)	
N3	0.08874 (14)	0.82597 (13)	0.45187 (11)	0.0148 (3)	
C11	0.02042 (18)	0.77691 (17)	0.40054 (15)	0.0179 (3)	
H11	−0.0646	0.8267	0.3805	0.022*	
C12	0.06960 (19)	0.65643 (17)	0.37590 (15)	0.0204 (3)	
H12	0.0185	0.6242	0.3400	0.024*	
C13	0.19391 (19)	0.58355 (17)	0.40415 (15)	0.0209 (3)	
H13	0.2300	0.5010	0.3875	0.025*	
C14	0.26469 (19)	0.63353 (17)	0.45721 (15)	0.0205 (3)	
H14	0.3502	0.5855	0.4776	0.025*	
C15	0.20934 (18)	0.75428 (17)	0.48022 (14)	0.0179 (3)	
H15	0.2578	0.7877	0.5171	0.022*	
N4	0.12228 (15)	1.12831 (14)	0.33344 (12)	0.0157 (3)	
C16	0.05298 (18)	1.21171 (16)	0.25660 (14)	0.0177 (3)	
H16	−0.0518	1.2052	0.2758	0.021*	
C17	0.1294 (2)	1.30722 (17)	0.14993 (15)	0.0215 (3)	
H17	0.0773	1.3660	0.0976	0.026*	
C18	0.2822 (2)	1.31577 (18)	0.12077 (16)	0.0246 (4)	
H18	0.3368	1.3805	0.0483	0.030*	
C19	0.3546 (2)	1.2278 (2)	0.19953 (17)	0.0263 (4)	
H19	0.4600	1.2304	0.1809	0.032*	
C20	0.27139 (18)	1.13681 (18)	0.30494 (15)	0.0211 (3)	
H20	0.3208	1.0781	0.3593	0.025*	
Cl1P	0.64390 (4)	0.40483 (4)	0.24420 (3)	0.01718 (10)	
O1P	0.53210 (15)	0.45637 (14)	0.33397 (13)	0.0305 (3)	
O2P	0.5964 (2)	0.44001 (15)	0.14139 (13)	0.0374 (4)	
O3P	0.66002 (17)	0.26002 (13)	0.29401 (13)	0.0304 (3)	
O4P	0.78435 (15)	0.46338 (17)	0.20959 (16)	0.0387 (4)	

### Atomic displacement parameters (Å^2^)

**Table:**

	*U*^11^	*U*^22^	*U*^33^	*U*^12^	*U*^13^	*U*^23^
Cr1	0.01311 (16)	0.01227 (16)	0.01356 (16)	0.00203 (12)	−0.00396 (13)	−0.00714 (12)
F1	0.0183 (4)	0.0153 (4)	0.0190 (4)	0.0016 (3)	−0.0062 (4)	−0.0100 (3)
N1	0.0146 (6)	0.0150 (6)	0.0150 (6)	0.0008 (5)	−0.0050 (5)	−0.0071 (5)
C1	0.0153 (7)	0.0167 (7)	0.0178 (7)	0.0020 (5)	−0.0060 (6)	−0.0086 (6)
C2	0.0142 (7)	0.0245 (8)	0.0184 (7)	0.0011 (6)	−0.0046 (6)	−0.0110 (6)
C3	0.0161 (7)	0.0263 (8)	0.0183 (7)	−0.0041 (6)	−0.0036 (6)	−0.0067 (6)
C4	0.0225 (8)	0.0195 (8)	0.0223 (8)	−0.0046 (6)	−0.0066 (7)	−0.0063 (6)
C5	0.0197 (7)	0.0157 (7)	0.0195 (7)	0.0000 (6)	−0.0065 (6)	−0.0081 (6)
N2	0.0149 (6)	0.0152 (6)	0.0149 (6)	0.0021 (5)	−0.0042 (5)	−0.0069 (5)
C6	0.0188 (7)	0.0180 (7)	0.0182 (7)	0.0018 (6)	−0.0054 (6)	−0.0085 (6)
C7	0.0196 (7)	0.0241 (8)	0.0227 (8)	0.0014 (6)	−0.0089 (7)	−0.0101 (6)
C8	0.0176 (7)	0.0234 (8)	0.0212 (8)	0.0047 (6)	−0.0068 (6)	−0.0075 (6)
C9	0.0198 (7)	0.0193 (7)	0.0230 (8)	0.0061 (6)	−0.0067 (7)	−0.0098 (6)
C10	0.0185 (7)	0.0170 (7)	0.0188 (7)	0.0022 (6)	−0.0053 (6)	−0.0083 (6)
Cr2	0.00999 (15)	0.01573 (17)	0.01295 (16)	0.00214 (12)	−0.00418 (12)	−0.00595 (12)
F2	0.0138 (4)	0.0204 (5)	0.0191 (4)	0.0021 (3)	−0.0077 (4)	−0.0080 (4)
N3	0.0133 (6)	0.0163 (6)	0.0135 (6)	0.0011 (5)	−0.0033 (5)	−0.0057 (5)
C11	0.0153 (7)	0.0199 (7)	0.0187 (7)	0.0013 (6)	−0.0056 (6)	−0.0076 (6)
C12	0.0190 (7)	0.0213 (8)	0.0225 (8)	0.0003 (6)	−0.0065 (6)	−0.0101 (6)
C13	0.0200 (7)	0.0180 (7)	0.0218 (8)	0.0023 (6)	−0.0035 (6)	−0.0084 (6)
C14	0.0172 (7)	0.0214 (8)	0.0198 (7)	0.0056 (6)	−0.0053 (6)	−0.0072 (6)
C15	0.0146 (7)	0.0220 (8)	0.0176 (7)	0.0033 (6)	−0.0055 (6)	−0.0087 (6)
N4	0.0140 (6)	0.0183 (6)	0.0149 (6)	0.0007 (5)	−0.0035 (5)	−0.0076 (5)
C16	0.0175 (7)	0.0193 (7)	0.0165 (7)	0.0023 (6)	−0.0056 (6)	−0.0077 (6)
C17	0.0266 (8)	0.0191 (7)	0.0178 (7)	0.0018 (6)	−0.0068 (7)	−0.0068 (6)
C18	0.0272 (9)	0.0224 (8)	0.0196 (8)	−0.0054 (7)	−0.0003 (7)	−0.0073 (6)
C19	0.0183 (8)	0.0327 (10)	0.0242 (8)	−0.0051 (7)	−0.0013 (7)	−0.0090 (7)
C20	0.0149 (7)	0.0275 (8)	0.0194 (7)	−0.0010 (6)	−0.0046 (6)	−0.0073 (6)
Cl1P	0.01680 (17)	0.01445 (17)	0.02051 (18)	0.00369 (12)	−0.00615 (14)	−0.00815 (13)
O1P	0.0234 (6)	0.0276 (7)	0.0369 (8)	0.0019 (5)	0.0019 (6)	−0.0201 (6)
O2P	0.0580 (10)	0.0308 (8)	0.0269 (7)	−0.0049 (7)	−0.0253 (7)	−0.0007 (6)
O3P	0.0427 (8)	0.0139 (6)	0.0382 (8)	0.0093 (5)	−0.0219 (7)	−0.0092 (5)
O4P	0.0178 (6)	0.0411 (8)	0.0597 (10)	−0.0049 (6)	−0.0017 (7)	−0.0291 (8)

### Geometric parameters (Å, º)

**Table:**

Cr1—F1^i^	1.8566 (10)	Cr2—N4	2.0799 (17)
Cr1—F1	1.8566 (10)	Cr2—N4^ii^	2.0800 (17)
Cr1—N1^i^	2.0867 (16)	Cr2—N3^ii^	2.0908 (14)
Cr1—N1	2.0867 (16)	Cr2—N3	2.0908 (14)
Cr1—N2^i^	2.0929 (15)	N3—C11	1.345 (2)
Cr1—N2	2.0929 (15)	N3—C15	1.350 (2)
N1—C1	1.3478 (19)	C11—C12	1.385 (2)
N1—C5	1.348 (2)	C11—H11	0.9500
C1—C2	1.386 (2)	C12—C13	1.383 (2)
C1—H1	0.9500	C12—H12	0.9500
C2—C3	1.384 (2)	C13—C14	1.386 (3)
C2—H2	0.9500	C13—H13	0.9500
C3—C4	1.396 (2)	C14—C15	1.385 (2)
C3—H3	0.9500	C14—H14	0.9500
C4—C5	1.386 (2)	C15—H15	0.9500
C4—H4	0.9500	N4—C16	1.341 (2)
C5—H5	0.9500	N4—C20	1.348 (2)
N2—C6	1.343 (2)	C16—C17	1.387 (2)
N2—C10	1.350 (2)	C16—H16	0.9500
C6—C7	1.388 (2)	C17—C18	1.382 (3)
C6—H6	0.9500	C17—H17	0.9500
C7—C8	1.383 (2)	C18—C19	1.391 (3)
C7—H7	0.9500	C18—H18	0.9500
C8—C9	1.386 (3)	C19—C20	1.378 (3)
C8—H8	0.9500	C19—H19	0.9500
C9—C10	1.381 (2)	C20—H20	0.9500
C9—H9	0.9500	Cl1P—O4P	1.4342 (15)
C10—H10	0.9500	Cl1P—O3P	1.4342 (14)
Cr2—F2^ii^	1.8552 (10)	Cl1P—O2P	1.4351 (15)
Cr2—F2	1.8552 (10)	Cl1P—O1P	1.4416 (14)
			
F1^i^—Cr1—F1	180.0	F2^ii^—Cr2—N4^ii^	90.36 (6)
F1^i^—Cr1—N1^i^	90.52 (5)	F2—Cr2—N4^ii^	89.64 (6)
F1—Cr1—N1^i^	89.48 (5)	N4—Cr2—N4^ii^	180.0
F1^i^—Cr1—N1	89.48 (5)	F2^ii^—Cr2—N3^ii^	90.31 (5)
F1—Cr1—N1	90.52 (5)	F2—Cr2—N3^ii^	89.69 (5)
N1^i^—Cr1—N1	180.00 (7)	N4—Cr2—N3^ii^	87.08 (6)
F1^i^—Cr1—N2^i^	90.40 (5)	N4^ii^—Cr2—N3^ii^	92.92 (6)
F1—Cr1—N2^i^	89.60 (5)	F2^ii^—Cr2—N3	89.68 (5)
N1^i^—Cr1—N2^i^	92.68 (6)	F2—Cr2—N3	90.32 (5)
N1—Cr1—N2^i^	87.32 (6)	N4—Cr2—N3	92.92 (6)
F1^i^—Cr1—N2	89.60 (5)	N4^ii^—Cr2—N3	87.08 (6)
F1—Cr1—N2	90.40 (5)	N3^ii^—Cr2—N3	180.0
N1^i^—Cr1—N2	87.32 (6)	C11—N3—C15	118.33 (14)
N1—Cr1—N2	92.68 (6)	C11—N3—Cr2	120.91 (11)
N2^i^—Cr1—N2	180.0	C15—N3—Cr2	120.55 (11)
C1—N1—C5	118.53 (14)	N3—C11—C12	122.38 (15)
C1—N1—Cr1	119.68 (11)	N3—C11—H11	118.8
C5—N1—Cr1	121.77 (11)	C12—C11—H11	118.8
N1—C1—C2	122.25 (15)	C13—C12—C11	119.25 (16)
N1—C1—H1	118.9	C13—C12—H12	120.4
C2—C1—H1	118.9	C11—C12—H12	120.4
C3—C2—C1	119.33 (15)	C12—C13—C14	118.63 (15)
C3—C2—H2	120.3	C12—C13—H13	120.7
C1—C2—H2	120.3	C14—C13—H13	120.7
C2—C3—C4	118.55 (15)	C15—C14—C13	119.30 (15)
C2—C3—H3	120.7	C15—C14—H14	120.4
C4—C3—H3	120.7	C13—C14—H14	120.4
C5—C4—C3	119.09 (16)	N3—C15—C14	122.11 (15)
C5—C4—H4	120.5	N3—C15—H15	118.9
C3—C4—H4	120.5	C14—C15—H15	118.9
N1—C5—C4	122.22 (15)	C16—N4—C20	118.68 (15)
N1—C5—H5	118.9	C16—N4—Cr2	120.86 (11)
C4—C5—H5	118.9	C20—N4—Cr2	120.25 (12)
C6—N2—C10	118.41 (14)	N4—C16—C17	122.13 (16)
C6—N2—Cr1	120.40 (10)	N4—C16—H16	118.9
C10—N2—Cr1	121.05 (11)	C17—C16—H16	118.9
N2—C6—C7	122.17 (15)	C18—C17—C16	119.13 (17)
N2—C6—H6	118.9	C18—C17—H17	120.4
C7—C6—H6	118.9	C16—C17—H17	120.4
C8—C7—C6	119.12 (16)	C17—C18—C19	118.76 (17)
C8—C7—H7	120.4	C17—C18—H18	120.6
C6—C7—H7	120.4	C19—C18—H18	120.6
C7—C8—C9	118.91 (16)	C20—C19—C18	119.08 (17)
C7—C8—H8	120.5	C20—C19—H19	120.5
C9—C8—H8	120.5	C18—C19—H19	120.5
C10—C9—C8	119.00 (15)	N4—C20—C19	122.20 (17)
C10—C9—H9	120.5	N4—C20—H20	118.9
C8—C9—H9	120.5	C19—C20—H20	118.9
N2—C10—C9	122.39 (16)	O4P—Cl1P—O3P	110.13 (10)
N2—C10—H10	118.8	O4P—Cl1P—O2P	109.84 (11)
C9—C10—H10	118.8	O3P—Cl1P—O2P	109.44 (9)
F2^ii^—Cr2—F2	180.0	O4P—Cl1P—O1P	109.02 (9)
F2^ii^—Cr2—N4	89.64 (6)	O3P—Cl1P—O1P	108.90 (9)
F2—Cr2—N4	90.36 (6)	O2P—Cl1P—O1P	109.50 (10)
			
C5—N1—C1—C2	1.2 (2)	C15—N3—C11—C12	−0.1 (2)
Cr1—N1—C1—C2	179.81 (12)	Cr2—N3—C11—C12	−175.00 (12)
N1—C1—C2—C3	0.3 (2)	N3—C11—C12—C13	−0.4 (3)
C1—C2—C3—C4	−1.2 (2)	C11—C12—C13—C14	0.4 (3)
C2—C3—C4—C5	0.5 (3)	C12—C13—C14—C15	0.0 (2)
C1—N1—C5—C4	−1.9 (2)	C11—N3—C15—C14	0.5 (2)
Cr1—N1—C5—C4	179.55 (13)	Cr2—N3—C15—C14	175.44 (12)
C3—C4—C5—N1	1.0 (3)	C13—C14—C15—N3	−0.5 (2)
C10—N2—C6—C7	0.4 (2)	C20—N4—C16—C17	−0.9 (2)
Cr1—N2—C6—C7	−175.43 (12)	Cr2—N4—C16—C17	173.85 (12)
N2—C6—C7—C8	−0.6 (3)	N4—C16—C17—C18	0.9 (2)
C6—C7—C8—C9	0.4 (3)	C16—C17—C18—C19	0.1 (2)
C7—C8—C9—C10	0.0 (3)	C17—C18—C19—C20	−1.2 (3)
C6—N2—C10—C9	0.1 (2)	C16—N4—C20—C19	−0.3 (2)
Cr1—N2—C10—C9	175.84 (12)	Cr2—N4—C20—C19	−175.00 (14)
C8—C9—C10—N2	−0.2 (3)	C18—C19—C20—N4	1.3 (3)

Symmetry codes: (i) −*x*, −*y*+2, −*z*; (ii) −*x*, −*y*+2, −*z*+1.

### Hydrogen-bond geometry (Å, º)

**Table:**

*D*—H···*A*	*D*—H	H···*A*	*D*···*A*	*D*—H···*A*
C1—H1···F1	0.95	2.36	2.892 (2)	115
C6—H6···F1	0.95	2.31	2.874 (2)	118
C11—H11···F2	0.95	2.32	2.879 (2)	117
C16—H16···F2	0.95	2.39	2.915 (2)	115
C14—H14···O1*P*	0.95	2.61	3.154 (2)	117
C9—H9···O2*P*	0.95	2.63	3.320 (3)	130
C1—H1···O3*P*^iii^	0.95	2.41	3.107 (2)	130
C4—H4···O1*P*^iv^	0.95	2.51	3.368 (2)	150
C5—H5···F1^i^	0.95	2.38	2.900 (2)	114
C10—H10···F1^i^	0.95	2.31	2.863 (2)	117
C15—H15···F2^ii^	0.95	2.29	2.856 (2)	117
C20—H20···F2^ii^	0.95	2.34	2.860 (2)	114
C15—H15···O3*P*^v^	0.95	2.64	3.466 (2)	146
C19—H19···O2*P*^vi^	0.95	2.58	3.227 (3)	125

Symmetry codes: (i) −*x*, −*y*+2, −*z*; (ii) −*x*, −*y*+2, −*z*+1; (iii) *x*−1, *y*+1, *z*; (iv) *x*−1, *y*, *z*; (v) −*x*+1, −*y*+1, −*z*+1; (vi) *x*, *y*+1, *z*.
